# Impact of a disability-targeted microentrepreneurship programme in Kenya: study protocol for a randomised controlled trial of the InBusiness programme

**DOI:** 10.1186/s13063-023-07848-2

**Published:** 2023-12-11

**Authors:** Lena Morgon Banks, Stella Kepha, Anja Zinke-Allmang, Paul M. Gichuki, Nathaniel Scherer, Charles Mwandawiro, Mark Carew, Doris Njomo, Shanquan Chen, Collins Okoyo, Calum Davey, Tom Shakespeare, Maria Antonella Pereira, Divya Goyal, Hannah Kuper

**Affiliations:** 1 International Centre for Evidence in Disability, London School of Hygiene & Tropical Medicine, London, UK; 2 Kenya Medical Research Institute, Nairobi, Kenya; 3 Georgetown University, Washington, DC USA

**Keywords:** Disability, Livelihood, Randomised controlled trial, Kenya, Microenterprise

## Abstract

**Background:**

There is a lack of evidence on the effectiveness of livelihood interventions amongst people with disabilities. In many countries, self-employment or microentrepreneurship is a dominant source of livelihoods for people with disabilities and their caregivers. However, this group may face heightened barriers to successful microentrepreneurship, including discrimination, exclusion from training or inaccessible transport, infrastructure and communication systems. The InBusiness programme is a livelihoods programme targeted to microentrepeneurs with disabilities or their caregivers delivered by a consortium of non-governmental organisations. The programme focuses on improving the skills, practices and opportunities of microentrepreneurs while linking them with procurement opportunities with private and public institutions. This protocol describes a randomised controlled trial of the InBusiness programme in eight counties of Kenya.

**Methods:**

The randomised controlled trial will involve 495 microentrepreneurs who have been verified as eligible for InBusiness by programme implementers. Individuals will be randomised within counties, either being invited to enrol in InBusiness in March 2023 or allocated to a control group. Participants in the control arm will receive information about compliance with business-related laws and available social protection programmes. The trial will assess the impact of InBusiness on household consumption and individual economic empowerment (primary outcomes) as well as food security, well-being, social attitudes, unmet need for disability-related services and microenterprise profits (secondary outcomes). Baseline was conducted in March 2023, and follow-up will be 24 months from baseline (12 months from completion of the programme). Analysis will be through intention to treat. A process evaluation will explore fidelity, mechanisms of impact and the role of context, and complementary qualitative research with participants will be used to triangulate findings across the trial.

**Discussion:**

This study will provide evidence on the impact of a large-scale disability-targeted livelihood programme on household and individual financial security and well-being. Currently, there is a lack of evidence on the effectiveness of livelihood programmes amongst people with disabilities, and so this trial can help inform the design and delivery of InBusiness as well as other livelihood programmes targeted to people with disabilities.

**Trial registration:**

ClinicalTrials.gov ISRCTN13693137. Registered on April 24, 2023.

**Supplementary Information:**

The online version contains supplementary material available at 10.1186/s13063-023-07848-2.

## Introduction

### Background and rationale

The 2022 Kenya Demographic and Health Survey estimates that almost 6% of the population of Kenya has a disability [1]. The Kenya Persons with Disabilities Act of 2003 and its 2012 revision defines disability as a “physical, sensory, mental or other impairment, including any visual, hearing, learning or physical incapability, which impacts adversely on social, economic or environmental participation” [2]. The true prevalence of disability is likely to be higher, as recent WHO estimates suggest that 16% of the world’s population are people with disabilities [3]. Both globally and in Kenya, people with disabilities are more likely to be living in poverty and excluded from livelihood opportunities compared to people without disabilities [1, 4, 5]. Improving access to decent work and alleviating poverty “for all” are key aims of the Sustainable Development Goals [6] and are in line with national policies in Kenya [2].

Self-employment, or microentrepreneurship, is a major source of livelihoods in low- and middle-income countries. In Kenya, there were approximately 7.41 million microenterprises in 2016, accounting for 34% of Kenya’s GDP and employing over 14.9 million people [7]. The participation of people with disabilities in Kenya in this sector is unclear, but if following international trends, it is likely to represent a significant livelihood source for people with disabilities due to widespread exclusion from waged employment [8, 9]. There are some social protection measures in Kenya to improve opportunities for microentrepreneurs with disabilities, such as the access to public procurement opportunities programme. This programme sets aside 30% of annual government procurement budget for businesses run by people with disabilities, youth and women, of which at least 2% is reserved for people with disabilities. However, this quota is often underfilled, with only 1.3% of the budget was awarded to firms owned by people with disabilities in 2018 [10].

People with disabilities and their caregivers may face additional barriers to successful microentrepreneurship, leading to job insecurity and low and unstable earnings [11]. For example, people with disabilities may lack skills and training from earlier exclusion from education [9]. Further, discrimination and poor informational, communication and infrastructure accessibility of linked systems (e.g. transport, places to buy inputs/sell outputs) can limit opportunities for growth. Meanwhile, caregivers of people with disabilities can face both opportunity and direct costs associated with caregiving, reducing time and resources available to invest in their microenterprises. Improving the management and profitability of microenterprises run by people with disabilities and caregivers of people with disabilities may help to increase their livelihoods and financial security. However, a recent systematic review found a lack of evidence on the impact of interventions designed to improve livelihoods for people with disabilities and their households, and available evidence was often of poor quality [12].

Consequently, this study will conduct a randomised controlled trial (RCT) of the InBusiness programme in Kenya. InBusiness is run by a consortium of non-governmental organisations (NGOs) — Light for the World (LftW), Humanity & Inclusion (HI) and Sense International (SI). The programme aims to improve the skills of microentrepreneurs with disabilities and microentrepreneurs who are caregivers of people with disabilities, through mentorship and classroom trainings. It also facilitates pathways for expanding their microenterprises, such as through increased procurement opportunities with public and private institutions (PPIs) and transfers of business-related assets. The programme is being implemented in eight counties in Kenya and has potential for wider scale-up in Kenya and other settings.

### Aims and objectives

The overall aim of the research is to assess the impact of InBusiness on improving social and economic wellbeing of people with disabilities and their households. Specific objectives include the following:

1. To assess the impact of InBusiness on household per capita expenditures and economic empowerment
2. To evaluate the impact of InBusiness on quality of life and access to key disability-related goods and services
3. To explore the aspects of InBusiness that were perceived to be most important for achieving impacts amongst participants
4. To examine barriers and enablers to the implementation and delivery of InBusiness from the perspective of participants and implementers.

## Methods and analysis

### Design

The study’s main design is an individual, superiority RCT of the LftW-led component of the InBusiness programme.^1^ It follows the SPIRIT guidelines for RCTs (Supplemental file 1). The LftW InBusiness programme is being implemented in 8 of 47 counties in Kenya. Individuals applying to InBusiness will be randomised to either the intervention or control group. The intervention group will be offered a place on the InBusiness programme for enrolment in March 2023. The control group will not be offered a place on the InBusiness programme but will receive information about compliance with business laws and on available social protection programmes. Baseline data collection will be conducted before randomisation and before participants are invited to enrol in InBusiness to minimise anticipatory behaviour. Follow-up will be conducted 24 months after baseline (12 months after the completion of the delivery of InBusiness).

The RCT will be complemented with a process evaluation and qualitative research. In-depth interviews will be conducted with participants in both study arms after the delivery of InBusiness is completed to explore their experiences of running a microenterprise, and for the intervention arm, and of participating in the InBusiness programme. In-depth interviews with programme implementers will also be conducted, and monitoring data from InBusiness and the endline of the RCT will be reviewed to explore the implementation fidelity, mechanisms of impact and contextual factors that can affect variations in outcomes. The process evaluation will use the Medical Research Council guidance for process evaluations of complex interventions as a framework [13].

### Eligibility criteria

Participants in both the control and intervention arms must meet InBusiness’ eligibility criteria. Eligibility for the InBusiness programme is based on the following: (1) Has a disability or is the caregiver of a person with a disability. Disability is defined as having a disability identification card issued by the national government (i.e. registered with the National Council for Persons with Disabilities), (2) lives and operates a microenterprise in one of the eight counties covered by the InBusiness programme, (3) has a business licence/permit or is in the process of applying for a permit, (4) has a microenterprise that has been in existence for at least 6 months, and (5) microenterprise has a monthly income of at least KES 10,000 (US $75). For past recruitments, these criteria have sometimes been relaxed slightly to obtain sufficient numbers of participants.

The identification process was conducted through a call for applications led by LftW, HI and SI. Calls for applications were distributed through Organisations of Persons with Disabilities (OPDs) through the umbrella OPD United Disabled Persons Kenya. Overall, 776 met programme eligibility.

### Recruitment and randomisation

Recruitment is through self-selection with LftW verification. LftW circulated calls to apply for the InBusiness programme in the eight counties where it will be operating in. Individuals submitted an application, and LftW verified if they meet eligibility criteria.

**Fig. 1:**
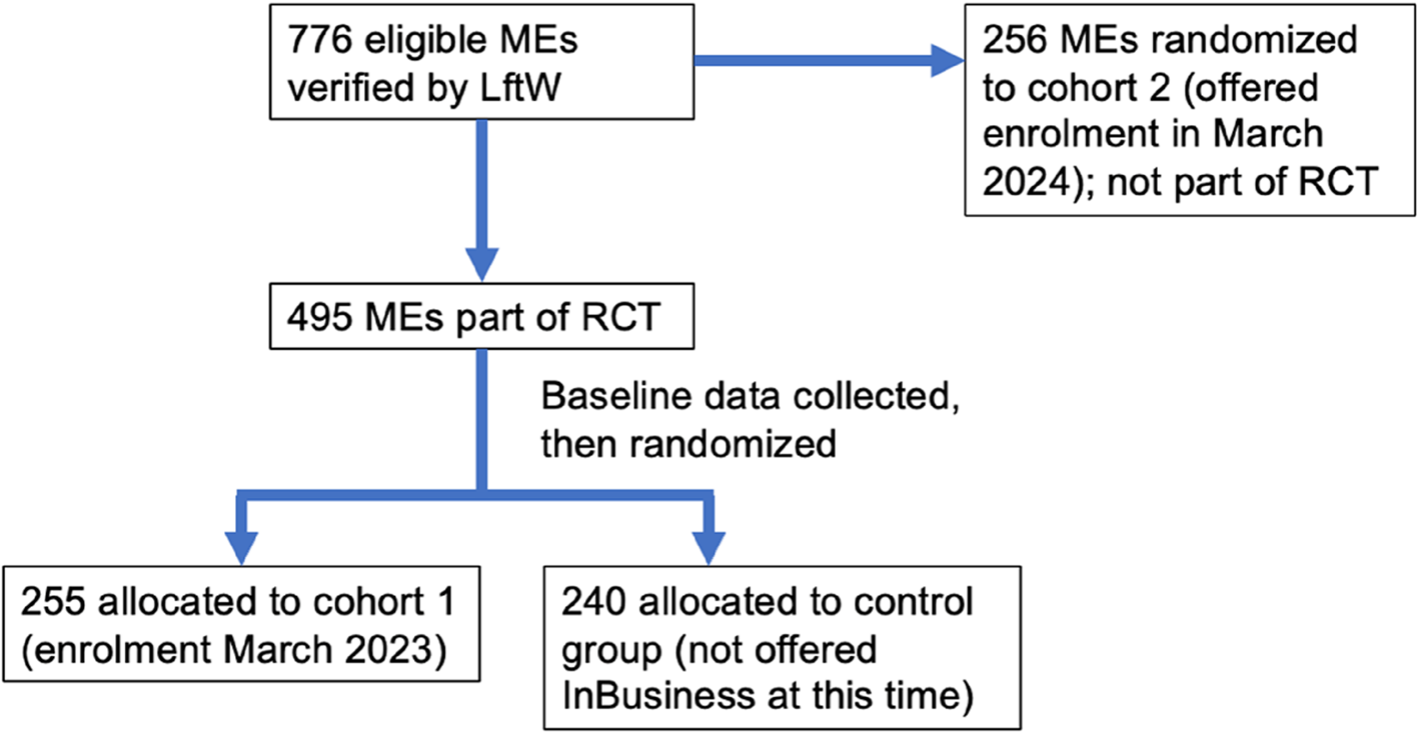
Flow diagram of recruitment and randomisation

LftW has funding to provide the programme to approximately 480 people across two cohorts (cohort 1: enrolment in March 2023 and cohort 2: enrolment in March 2024). LftW verified 776 eligible people between December 2022-February 2023. Within this group, individuals will be randomised to either cohort 1, cohort 2 or the control group (Fig. 1). The RCT will focus on cohort 1 (intervention group) and the control group. Cohort 2 will not be part of the RCT as they will not have been involved in the project for sufficient time at follow-up.

Randomisation will take place in two phases. Individuals will first be randomised to either cohort 2 or the group taking part in the RCT. Baseline data will then be collected from the RCT group, after which individuals will be randomised to cohort 1 or the control group. Randomisation will be within counties based on quotas from LftW on the number of placements available for cohorts 1 and 2, and each group will be roughly balanced by gender. Randomisation will be done through R by staff at the London School of Hygiene & Tropical Medicine (LSHTM).

If there are a small number of dropouts in people randomised to cohort 2 (< 20 individuals), LftW can recruit from the control group to avoid needing to re-advertising and re-verifying applicants. In this instance, individuals moving from control to cohort 2 will be selected randomly and will be excluded from the analysis.

For the qualitative research and process evaluation, 20–25 participants will be recruited from the RCT group prior to endline (predominantly people in cohort 1, with some from the control arm). Recruitment will be purposive to maximise heterogeneity by gender, impairment type, location, type of microenterprise and if the microentrepreneur (ME) is a caregiver or a person with a disability. Additionally, 15–20 InBusiness programme implementers and key partners will be selected based on their involvement in different stages of InBusiness design and delivery.

### Intervention

The InBusiness programme seeks to improve the social and economic wellbeing of MEs with disabilities and their households. The programme primarily targets people with disabilities who are the primary owner of a microenterprise but in a minority of cases will offer the programme to businesses run jointly by a people with disabilities and a caregiver or businesses run only by a caregiver of an adult/child with a disability. The programme provides business, technical, self-advocacy and compliance skills training and in-kind transfers of assets required to expand the business named “business growth kits” and facilitates procurement opportunities with PPIs.

The InBusiness programme will be delivered over 10 months. The core components include the following:

1. Classroom-based trainings: Microentrepreneurs will receive classroom training on skills to improve their business practices. Content covered in trainings includes record keeping, compliance with national and local regulations and self-advocacy.
2. Capacity building: One-to-one follow-up with microentrepreneurs to monitor their progress and implementation of trainings and to offer tailored advice to support their businesses.
3. Business growth kits: Microentrepreneurs will be provided with an in-kind asset to grow or improve the profitability of their business and/or support their participation in the business (e.g. assistive products). The asset will be tailored based on the needs of each microentrepreneur’s business and/or accessibility needs.
4. PPI disability inclusion training: PPIs will be trained on disability inclusion and encouraged to offer procurement opportunities to people with disabilities (e.g. through the access to public procurement opportunities programme).
5. Linking to procurement opportunities: Microentrepreneurs will be made aware of and supported to apply for procurement opportunities with local PPIs.

### Usual care

Microentrepreneurs in the control arm will receive usual care, meaning they will not be offered enrolment to the InBusiness programme during the duration of the trial. However, they are free to use any services or programmes operating in their area. They will be compensated for their participation in the study and offered information about available social protection programmes (e.g. procurement quotas for people with disabilities) and on compliance with local/national laws related to microenterprises.

### Data collection

Data for the RCT will be collected using questionnaires completed by participants in the intervention and control arms at baseline and endline. Data collection will be conducted in a place of the participants’ choosing (e.g. home, business). The questionnaire is based upon standard modules and from modules used in other studies (Table 1). It includes sections on the following: household composition, housing conditions, food security, savings and expenditures; individual economic empowerment, well-being and access to services; and microenterprise revenues, costs and practices. It was reviewed by programme implementers and piloted with individuals who had previously participated in the InBusiness programme to check for acceptability and understanding before full-scale data collection. The survey will take approximately 1 h to complete and will be delivered by trained enumerators using Open Data Kit (ODK).

**Table 1:** Outcome indicators for the RCT of the InBusiness programme

Outcome indicator	Description	Source
*Primary outcomes*		
Household consumption	Overall per capita household consumption and domains of expenditures (food, health) adjusted for inflation	Adapted version of [14]
Economic empowerment	Score on a 14-item financial self-efficacy tool	Adapted version of [15]
*Secondary outcomes*		
Subjective wellbeing	Total score on a 7-item tool on self-reported well-being	Adapted version of [16]
Food security	Proportion classified as facing moderate or severe food insecurity	Food Insecurity Experience Scale [17]
Social attitudes	Total score on 9-item tool on social attitudes	World Health Model Disability Survey [18]
Unmet need for disability-related goods and services	Self-reported unmet need for disability-related healthcare, assistive devices or assistance	Tool developed for study
Microenterprise profits	Net of revenues minus costs, adjusted for inflation	Tool developed for study

In-depth interviews will be conducted with 20–25 participants of the RCT at endline and with 15–20 programme implementers as part of the process evaluation. Interviews will use semi-structured topic guides and will be conducted by experienced researchers. They will be recorded, transcribed and, where needed, translated to English. Interviews with RCT participants will focus on barriers and enablers to running microenterprises. The InBusiness programme participants will also be asked about the perceived impact of the programme, the strengths and areas for improvement in different components to the programme and any recommendations for adapting the overall programme. Interviews with implementers will focus on their experience developing and/or delivering the components of InBusiness they were responsible for, including any challenges, adaptations and suggestions for improvements.

### Outcomes

A theory of change was created in collaboration with programme implementers to identify anticipated outcomes of the InBusiness programme. The ultimate aim of InBusiness is to improve the livelihoods of microentrepeneurs of people with disabilities and their households, which can lead to reduced poverty and improved well-being. The primary outcome measure is changes in household expenditures and economic empowerment (Table 1). Secondary outcome measures will include subjective well-being, food security, social attitudes, unmet need for disability-related goods and services and microenterprise profits. Indicators are tied to the existing tools where possible.

The endline survey will also collect information from the intervention arm about their experience participating in the programme, including the following: (a) details on receipt of different components of the intervention to explore fidelity and uptake (e.g. what was received, frequency, how delivered), (b) satisfaction with the programme overall and with specific components, (c) challenges experienced during any component of the intervention, and (d) self-reported impacts of participation.

### Sample size

One of primary outcome measures is household expenditure per capita. Using data from the Kenya Integrated Household Budget Survey (KIHBS) 2015/2016 [19], we assume an average household per capita expenditures of 7811 KSH. Standard deviation was not presented in the report, but we assume standard deviation of 40% of the mean (3124 KSH). With power of 80% and type 1 error of 5%, we would need a total sample size of 398 (199 per arm) to detect an effect change of 10% of household expenditure per capita. Assuming a loss to follow-up of 20% and a potential switch of 20 individuals from control group to cohort 2 (excluded from RCT analysis), we will need to recruit at least 497 individuals.

### Timeline

Baseline data collection is being conducted in March 2023, after verification but before randomisation and notification of participants on their allocation. Delivery of the intervention will be over 10 months beginning April 2023. Endline will be conducted 24 months post baseline (March 2024). The process evaluation and qualitative research will be conducted shortly after completion of the delivery of InBusiness, in January-March 2024. A description of activities and timelines is in Table 2.

**Table 2:** Schedule of enrolment, interventions and assessments

	**Study period**							
	**Enrolment**	**Allocation**	**Post-allocation (10 months)**					**Endline**
**Timepoint**	***-t***_***1***_	**0**	***2***	***4***	***6***	***8***	***10***	***24 months***
**Enrollment**								
** Eligibility screen**	X							
** Informed consent**	X							
** Allocation**		X						
**Interventions**								
*** Classroom training***			X	X	X	X	X	
*** Capacity building***			X	X	X	X	X	
*** Business growth kits***			X	X	X	X	X	
*** PPI trainings***			X	X	X	X	X	
*** Procurement***			X	X	X	X	X	
**Assessments**								
*** Baseline survey***	X	X						
*** Endline survey***								X
*** Process evaluation***							X	

### Data analysis

A detailed analysis plan will be published before the endline survey. The analysis will estimate intention-to-treat (ITT) effects.

We will assess the quality of the balance achieved by randomisation by describing the arms of the trial at baseline in terms of the primary and secondary outcomes and sociodemographic variables. If there is evidence of imbalance, based on subjective interpretation of the magnitude of the difference, then we will plan a priori to include such variables in the main analysis of the primary and secondary outcomes.

We will estimate the effect of the intervention by comparing the proportions (e.g. unmet need for disability-related goods and services) and the means/medians (e.g. household expenditures, economic empowerment score) between the arms of the trial. We will report the unadjusted estimated effects as risk ratios for binary outcomes and difference in the means for continuous outcomes. In the final analysis, to increase the precision of the estimates and reduce the risk of bias from imbalances at baseline, we will use regression to adjust for the baseline levels of the outcome, stratification variables and variables considered to be imbalanced at baseline. For binary outcomes, we will model the risk ratio with a modified Poisson regression [20]. For continuous outcomes, we will use linear regression. Where sufficient power allows, we will also disaggregate by ME characteristics such as gender and disability type.

Transcripts of in-depth interviews from RCT participants and programme implementers will be coded using NVivo 12 and analysed thematically [21]. Coding frameworks will be developed using the semi-structured interview guides as a starting point and additional codes included iteratively. Comparisons and interrelationships between themes and sub-groups (e.g. by gender, impairment type) will be conducted throughout the analysis. The process evaluation will also use data from the endline RCT and monitoring data from LftW on the receipt and user experience of different components of InBusiness. This data will be tabulated, and regressions used to explore differences amongst InBusiness participants (e.g. by gender, location, impairment type).

### Ethics and dissemination

Ethical approval has been received from the Institutional Review Boards at the London School of Hygiene and Tropical Medicine (UK) and the AMREF Health Africa Ethics and Scientific Review Committee (Kenya).

Written informed consent will be sought from all participants by experienced data collectors who underwent a 1-week training on study protocols, immediately prior to administering the survey or beginning an interview. All participants will be over 18 years of age, though some may have mild/moderate intellectual impairments that affect their ability to give true informed consent. Capacity to consent amongst all participants will be assessed through an “Evaluation to Sign Consent” [22], which asks participants five questions about their understanding of the information sheet. Participants who are unable to provide a satisfactory response even with clarifications will be deemed unable to provide true informed consent. In these instances, parents/guardians will provide their consent, and the participant will provide assent if they are still able to participate in data collection. Adaptations will be in place to support the direct participation of people with different impairments (e.g. sign language interpretation, simplified interview schedules and information sheets).

Data from participants will be anonymised, with the exception that programme implementers may be identified by job title unless requested otherwise during the informed consent process. Data storage and management protocols are governed by a Data Protection Impact Assessment and data management plan. Data will not be made publicly available as it would not be possible to provide a minimum dataset that would allow for reproduction of findings without possible identification of participants.

No specific discomfort, distress or hazards are expected as a result of any component of the research (participating in survey and/or in-depth interviews). Participants may feel uncomfortable discussing their experiences but will be reminded that they have the right to stop or refuse to answer any questions at any time, for any reason. Participants (with the exception of programme implementers) will receive a one-off payment of KSH 500 to compensate for their time and other costs linked to participating in the research.

LftW has in place separate monitoring procedures to report and address any harms arising from participation in the InBusiness programme. LSHTM takes primary responsibility for the design of the study and ensuring it meets appropriate standards. KEMRI will lead the data collection and ensure that it meets ethical requirements. KEMRI and LSHTM will be in regular conduct regarding the running of the trial, including on data collection, data analysis and dissemination of findings. Any concerns or instances of misconduct related to participating in the research (i.e. survey, in-depth interview) can be reported to the LSHTM Research Governance and Integrity Office (rgio@lshtm.ac.uk) or the Scientific Ethics Review Unit at KEMRI (seru@kemri.go.ke), which is separate from the research team. Annual reports on study progress will be reviewed by the LSHTM Ethics Committee. LftW is responsible for the delivery of InBusiness and for monitoring and addressing any harms that result from participation in the InBusiness programme. The trial team and LftW will meet quarterly to discuss the delivery of the InBusiness programme and the trial conduct.

The study funder (United Kingdom Foreign, Commonwealth and Development Office) will not be involved in data collection or management, analysis or publication decisions. LftW and other programme implementers were consulted during the study design, to ensure that trial outcomes were in line with the intended outcomes of the InBusiness programme. They will also be consulted to discuss preliminary findings, but ultimate decisions on if and what to publish will be made by the research team. Authorship on any papers or reports will be determined according to standard guidance [23]. Any significant protocol modifications will be updated within the trial registry [23].

Findings from the research will be disseminated to academic and nonacademic audiences through journal articles, short reports/infographics with main findings and in-person and online events. The initial findings will be shared back and verified with participants through in-person events in the counties where the trial took place.

## Discussion and impact

There are some limitations of this evaluation. First, only single masking (of individuals conducting analyses for RCT) is possible in an intervention of this nature. To minimise bias, we have stressed in the informed consent process that responses will not impact the receipt of any services and have chosen several outcome measures that are less subjective (e.g. household consumption). Second, some impacts of InBusiness may not be apparent in the 2-year follow-up period and require a longer time period to develop. We have mitigated this risk by choosing indicators that the research team and the programme implementers felt are likely to show positive change if the programme’s theory of change is correct. Finally, it is possible that some impacts, particularly sub-group analyses by gender and other characteristics, may be underpowered.

Still, InBusiness is an important programme to evaluate for several reasons. The InBusiness programme is a large-scale intervention that is being delivered across eight counties across Kenya, with potential for scale-up in the future. Data on the effectiveness of InBusiness can therefore build a case for continued investment in the programme — including potential adaptations and expansion to other context — and/or indicate areas through which to improve the programme. Further, this study on the impact of the InBusiness programme will be one of the few trials of a livelihoods intervention that targets people with disabilities and caregivers of people with disabilities [12]. Findings from this research therefore have the potential to inform the design and delivery of not only the InBusiness programme but also other disability-inclusive livelihood programmes. Governments, NGOs and other actors have limited resources to invest in programmes such as InBusiness, particularly given cuts to foreign aid and rising cost of living crises. The lack of evidence on the effectiveness of livelihood interventions amongst people with disabilities hinders informed policymaking and planning, a gap which can begin to be filled through evaluations of promising programmes such as InBusiness.

### Trial status

This is protocol version 2, which has been accepted by Ethics Committees at the London School of Hygiene & Tropical Medicine, Kenya Medical Research Institute and AMREF Health Africa Ethics and Scientific Review Committee (Kenya). Participant recruitment began on March 6, 2023, and ended March 24, 2023. This protocol paper was originally submitted during participant recruitment, but the resubmission with clarifications was treated as a new submission.

### Supplementary Information

**Additional file 1.** SPIRIT checklist.

## Data Availability

The dataset is not possible to anonymise in a way that would allow for reproduction of findings without identifying participants.

## Footnotes

1 HI and SI are running smaller adaptations of the programme for people in humanitarian settings and for people with deafblindness, respectively, but their samples are too small for inclusion in the RCT.
